# Supplementary material for: Sepsis-Induced Coagulopathy Phenotype Induced by Oxidized High-Density Lipoprotein Associated with Increased Mortality in Septic-Shock Patients
Source: Antioxidants (Basel). 2023 Feb 21;12(3):543. doi: 10.3390/antiox12030543 (PMC10045333; doi:10.3390/antiox12030543)

# SUPPLEMENTAL INFORMATION

## Sepsis-induced coagulopathy phenotype induced by oxidized high-density lipoprotein associated with increased mortality in septic-shock patients.

Yolanda Prado[1,2], Pablo Tapia[3], Felipe Eltit[4,5], Cristian Reyes-Martínez[6], Carmen G. Feijóo[6], Felipe Marchant[1,2], Claudia A. Riedel[2,7], Claudio Cabello-Verrugio[2,8,9], Jimmy Stehberg[10,*] and Felipe Simon[1,2,11,*]

[1]Laboratory of Integrative Physiopathology, Faculty of Life Sciences, Universidad Andres Bello, Santiago, Chile.
[2]Millennium Institute on Immunology and Immunotherapy, Santiago, Chile.
[3]Unidad de Paciente Crítico Adulto, Hospital Clínico La Florida, La Florida, Santiago, Chile.
[4]Department of Urologic Sciences, University of British Columbia, Vancouver, Canada.
[5]Vancouver Prostate Centre, Vancouver, Canada.
[6]Fish Immunology Laboratory, Faculty of Life Science, Universidad Andres Bello, Santiago, Chile.
[7]Laboratory of Endocrinology-Immunology, Faculty of Life Sciences, Universidad Andres Bello, Santiago, Chile.
[8]Laboratory of Muscle Pathology, Fragility and Aging, Faculty of Life Sciences, Universidad Andres Bello, Santiago, Chile.
[9]Center for the Development of Nanoscience and Nanotechnology (CEDENNA), Universidad de Santiago de Chile, Santiago, Chile.
[10]Laboratory of Neurobiology, Institute of Biomedical Sciences, Faculty of Medicine and Faculty of Life Science, Universidad Andres Bello, Santiago, Chile.
[11]Millennium Nucleus of Ion Channel-Associated Diseases (MiNICAD), Santiago, Chile.

### SUPPLEMENTAL MATERIAL AND METHODS

#### Determination of coagulation and blood flow changes induced by oxHDL in zebrafish

Adult zebrafish were maintained at the fish facility of the Universidad Andres Bello, following standard protocols. Wild type AB and Tg(fli1:eGFP)$^{y1}$ embryos were obtained by natural spawning and maintained at 28°C in E3 medium (5 mM NaCl, 0.17 mM KCl, 0.33 mM $CaCl_2$, 0.33 mM $MgSO_4$, pH 7.0). Larval ages are expressed in days post fertilization (dpf). Handling procedures were approved by the Commission of Bioethics and Biosafety of Universidad Andres Bello (N° 004/2021). Before microinjection, wild type and Tg(fli1:eGFP)$^{y1}$ 4 dpf larvae were anesthetized with 0.03% tricaine (Sigma, USA) and mounted in 1 % low melting agarose (Sigma, USA). Using a microneedle attached to an air-driven Cell Tram (NARISHIGE, Japan), larvae were non-injected or individually microinjected in the heart with 8 nL containing saline buffer, 75 ng HDL, 75 ng oxHDL and maintained at 28°C for 24 h in E3 medium. Non-treated larvae were used to confirm that the vehicle did not have adverse effects. To determine the existence of coagulation, wild type larvae were stained with o-dianisidine (0.6 mg/ml, 0.01 M sodium acetate, pH 5.2, 0.65% $H_2O_2$, and 40% ethanol) for 30 min at RT, washed with distilled water and fixed in 4% PFA at 4°C ON. Then, larvae were imaged with a Leica MZ12.5 stereomicroscopes and images were processed using ImageJ software and BioVoxxel plugin to quantify the total pixel intensity (IU) in a defined region of interest (ROI). To determine changes in blood flow, time-lapse assay were performed on 5 dpf Tg(fli1:eGFP)$^{y1}$

larvae using a Leica TCS Sp8 confocal microscope acquired imagines every 2 seconds for 60 seconds in the segment of the caudal vein above the midgut. Images were processed using ImageJ software.

## SUPPLEMENTAL TABLE LEGEND

**Supplemental Table S1. Demographic characteristics and clinical data of ICU SSP and NSSP, and healthy volunteers.**

## SUPPLEMENTAL FIGURE LEGEND

**Supplemental Figure S1. Plasma level of aPPT and correlation with oxHDL and INDEX in SSP.** Plasma level of aPPT (A). Statistical differences were assessed by student's t-test (Mann-Whitney) for SSP and NSSP. *$p < 0.05$. Correlation analyses between: aPPT with high-oxHDL (B, upper-left panel, $r = -0.52$; $p$ = NS), low-oxHDL (B, lower-left panel, $r = -0.8695$; $p = 0.0005$), high-INDEX (B, upper-right panel, $r = 0.5094$; $p$ = NS) and low-INDEX (B, lower-right panel, $r = -0.1357$; $p$ = NS).

**Supplemental Figure S2. Administration of oxHDL induces coagulation in zebrafish vasculature.** *Zebrafish larvae* were subjected to o-dianisidine staining to evaluate *in vivo* coagulation. Control (non-injected) or injected with saline solution (10 nL NaCl 0,09%), oxHDL (10 nL oxHDL 4 µg/µL) and HDL (10 nL HDL 4 µg/µL) for 24 h (A). Thrombus formation was analyzed 24 h post injection in the caudal vein by o-dianisidine staining. (B-E) Representative images of zebrafish larvae non-injected (B) or injected with saline solution (C), oxHDL (D) and HDL (E). Doted red box depicts o-dianisidine staining. (F) Quantification of o-dianisidine staining in caudal vein of *Zebrafish larvae* in control non-injected condition (dark grey circles) or injected with saline solution (grey circles), oxHDL (orange circles) and HDL (green circles). Results of the total pixel intensity (I.U.) in a defined region of interest (ROI), were normalized with the median value of control condition. Tg(fli1:eGFP)$^{y1}$ *larvae*, having the vasculature and thrombocytes fluorescently green labeled, were subjected to time lapse analysis to evaluate blood flow *in vivo* coagulation. Blood flow time lapse analysis was determined as the number of platelets observed in 60 seconds in a section of the caudal vein (doted red box) were performed by time lapse analysis, in non-injected or injected with saline solution, oxHDL, and HDL (G). (H-K). Representative images of Tg(fli1:eGFP)$^{y1}$ larvae non-injected (H) or injected with saline solution (I), oxHDL (J) and HDL (K). (L) Quantification of blood flow time lapse analysis in a section of the caudal vein of Tg(fli1:eGFP)$^{y1}$ *larvae* in control non-injected condition (dark grey circles) or injected with saline solution (grey circles), oxHDL (orange circles) and HDL (green circles). Statistical differences were assessed by a one-way analysis of variance (ANOVA) (Kruskal–Wallis) followed by Dunn's *post hoc* test. ***$p < 0.001$. Values are expressed as the median ± 10-90 percentile.

**Supplemental Figure S3. Plasma level of coagulation factors and soluble platelet adhesion proteins correlates with ISTH-DIC score in SSP.** Correlation analyses between: TP with ISTH-DIC score from high-oxHDL (A, upper panel $r = 0.7276$; $p = 0.0048$), and from high-INDEX (A, lower panel, $r = 0.801$; $p = 0.0018$), TAFI with ISTH-DIC score from high-oxHDL (B, upper panel, $r = 0.411$; $p = 0.16$), and from high-INDEX (B, lower panel, $r = 0.716$; $p = 0.008$), t-PA with ISTH-DIC score from high-oxHDL (C, upper panel, $r = -0.2624$; $p = 0.3865$), and from high-INDEX (C, lower panel, $r = -0.6086$; $p = 0.0357$), TFPI with ISTH-DIC score from high-oxHDL (D, upper panel, $r = -0.645$; $p = 0.017$), and from high-INDEX (D, lower panel, $r = -0.541$; $p = 0.048$), svWF with ISTH-DIC score from high-oxHDL (E, upper panel, $r = 0.6186$; $p = 0.0242$), and from high-INDEX (E, lower panel, $r = 0.779$; $p = 0.002$), sP-Sel with ISTH-DIC score from high-oxHDL (F, upper panel, $r = 0.5797$; $p = 0.038$), and from high-INDEX (F, lower panel, $r = 0.709$; $p = 0.01$).

**Supplemental Figure S4. Coagulation factors and soluble platelet adhesion proteins expression in CMEC form SSP.** CMEC were magnetic bead-based immunoseparated from HV, high-oxHDL and low-oxHDL SSP blood samples and protein expression analyses by flow cytometry were performed.

**Supplemental Figure S5. Coagulation factors and soluble platelet adhesion proteins expression in CMEC correlates with ISTH-DIC score in SSP.** Correlation analyses between: TP with ISTH-DIC score from high-oxHDL (A, middle-left panel, $r = 0.5399$; $p = 0.047$) and from high-INDEX (A, right panel, $r = 0.647$; $p = 0.023$), TAFI with ISTH-DIC score from high-oxHDL (B, middle-left panel, $r = 0.2989$; $p = 0.03212$) and from high-INDEX (B, right panel, $r = 0.607$; $p = 0.036$), t-PA with ISTH-DIC score from high-oxHDL (C, middle-left panel, $r = –0.359$; $p = 0.228$) and from high-INDEX (C, right panel, $r = –0.565$; $p = 0.0445$), TFPI with ISTH-DIC score from high-oxHDL (D, middle-left panel, $r = –0.333$; $p = 0.267$) and from high-INDEX (D, right panel, $r = –0.519$; $p = 0.044$), vWF with ISTH-DIC score from high-oxHDL (E, middle-left panel, $r = 0.554$; $p = 0.049$) and from high-INDEX (E, right panel, $r = 0.7176$; $p = 0.009$), P-Sel with ISTH-DIC score from high-oxHDL (F, middle-left panel, $r = 0.338$; $p = 0.259$) and from high-INDEX (F, right panel, $r = 0.6946$; $p = 0.0122$).

**Supplemental Figure S6. Coagulation factors and platelet adhesion proteins expression in cultured EC exposed to plasma from SSP.** Plasma samples were collected from HV, high-oxHDL and low-oxHDL SSP blood samples and then added to cultured EC for 24 h.

**Supplemental Figure S7. Coagulation factors and platelet adhesion proteins expression in cultured EC exposed to plasma from SSP correlates with ISTH-DIC score in SSP.** Correlation analyses between: TP with ISTH-DIC score from high-oxHDL (A, middle-left panel, r = 0.3929; p = 0.1841) and from high-INDEX (A, right panel, r = 0.665; p = 0.018), TAFI with ISTH-DIC score from high-oxHDL (B, middle-left panel, r = 0.2989; p = 0.3212) and from high-INDEX (B, right panel, r = 0.607; p = 0.036), t-PA with ISTH-DIC score from high-oxHDL (C, middle-left panel, r = –0.4951; p = 0.085) and from high-INDEX (C, right panel, r = –0.672; p = 0.017), TFPI with ISTH-DIC score from high-oxHDL (D, middle-left panel, r = –0.641; p = 0.018) and from high-INDEX (D, right panel, r = –0.575; p = 0.041), vWF with ISTH-DIC score from high-oxHDL (E, middle-left panel, r = 0.2581; p = 0.3946) and from high-INDEX (E, right panel, r = 0.6049; p = 0.0372), P-Sel with ISTH-DIC score from high-oxHDL (F, middle-left panel, r = 0.516; p = 0.0411) and from high-INDEX (F, right panel, r = 0.7371; p = 0.0062).

**Supplemental Figure S8. Coagulation factors and platelet adhesion proteins mRNA expression in cultured EC exposed to exogenous SSP plasma preparation.**

## SUPPLEMENTAL MOVIE LEGENDS

**Supplemental Movie S1.** Representative time lapse in control non-injected condition. Representative time lapse of Tg(fli1:eGFP)$^{y1}$ 4 dpf larvae non-microinjected in the heart with 8 nL of saline buffer (NaCl 0,9%)

**Supplemental Movie S2.** Representative time lapse in saline-injected condition. Representative time lapse of Tg(fli1:eGFP)$^{y1}$ 4 dpf larvae microinjected in the heart with 8 nL of saline buffer (NaCl 0,9%)

**Supplemental Movie S3.** Representative time lapse in oxHDL-injected condition. Representative time lapse of Tg(fli1:eGFP)$^{y1}$ 4 dpf larvae microinjected in the heart with 8 nL of oxHDL (75 ng).

**Supplemental Movie S4.** Representative time lapse in HDL-injected condition. Representative time lapse of Tg(fli1:eGFP)$^{y1}$ 4 dpf larvae microinjected in the heart with 8 nL of oxHDL (75 ng).

# Supplemental Table S1

**Supplemental Table S1.**
**Demographic characteristics and clinical data of UCI SSP and NSSP, and healthy volunteers**

| Variable | Healthy Volunteers (n=39) | UCI SSP (n=26) | UCI NSSP (n=16) |
|---|---|---|---|
| Mortality at 30 days, % | 0 | 46,2 | 50 |
| APACHE II, median (IQR) | ND | 23.5 (20.5-25) | 25 (23-27) |
| SOFA, median (IQR) | ND | 12 (9-13) | 10 (9-13) |
| Age (ys), median (IQR) | 60 (42-70) | 64 (53-76) | 65 (55-73.5) |
| BMI, median (IQR) | 24.8 (22.9-29.5) | 26.1 (23.8-30.2) | 27.4 (25.3-28.7) |
| Male sex, (% of male) | 54 | 54 | 75 |
| Time of sampling (h)*, median (IQR) | NA | 55 (50-72) | 61 (49-70) |
| CRP (mg/dl), median (IQR) | ND | 30,4 ( 24.8-32.4) | 25.4 ( 19.2-27.8) |
| Resuscitation Fluid (Liter),median (IQR) | NA | 6.1 (5.4 -7.4) | 6.0 (5.2-7.6) |
| Blood lactate level (mmol/L)median (IQR) | ND | 9.1 (7.2-13.9) | 8.7 (6.6-13.5) |
| Norepinephrine (ug/kg/min) median (IQR) | NA | 0.31 (0.22-0.59) | 0.24 (0.18-0.57) |
| Renal replacement therapy*(%) | 0 | 28,2 | 25 |
| Corticosteroids, % | NA | 81 | 69 |
| Mechanical ventilation, % | 0 | 100 | 100 |

**Definition of abbreviations:**
BMI, body mass index; CRP, C-reactive protein; APACHE-II,
Acute Physiology and Chronic Health Evaluation II score; SOFA, Sepsis-related Organ Failure Assessment,
IQR: interquartile range (expressed as percentile 25th, percentile 75th), ND: non-determined, NA_ non-applicable
* The sample for analysis was collected always prior to connection to a renal replacement therapy.

# Supplemental Figure S1

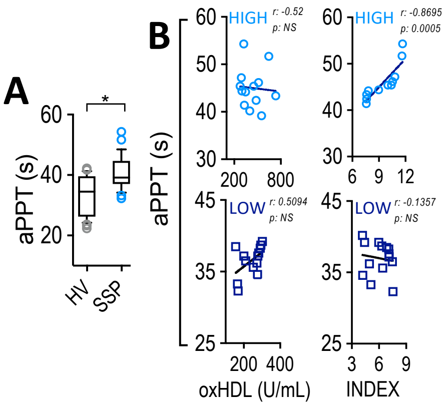

# Supplemental Figure S2

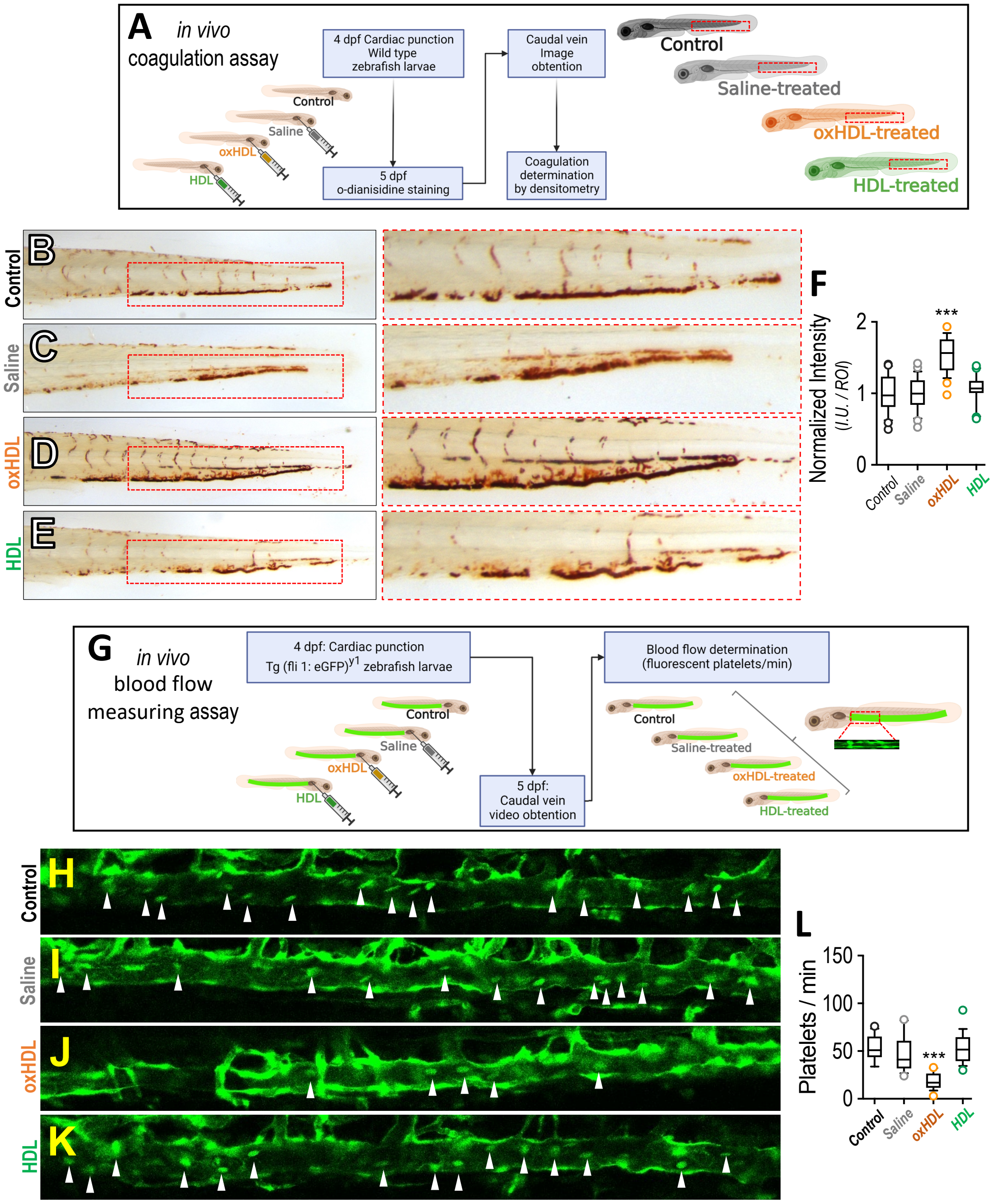

# Supplemental Figure S3

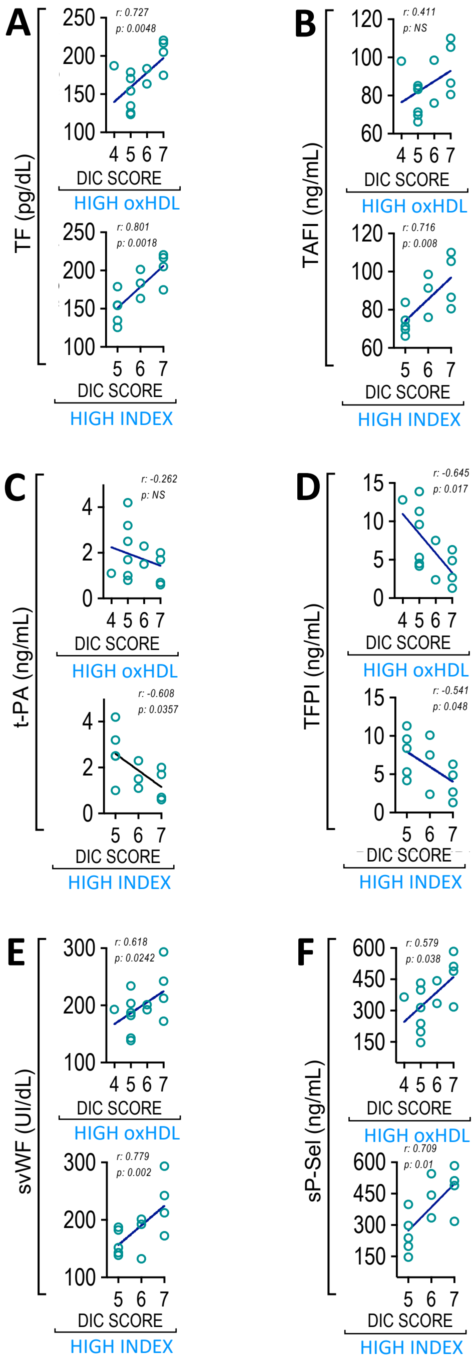

# Supplemental Figure S4

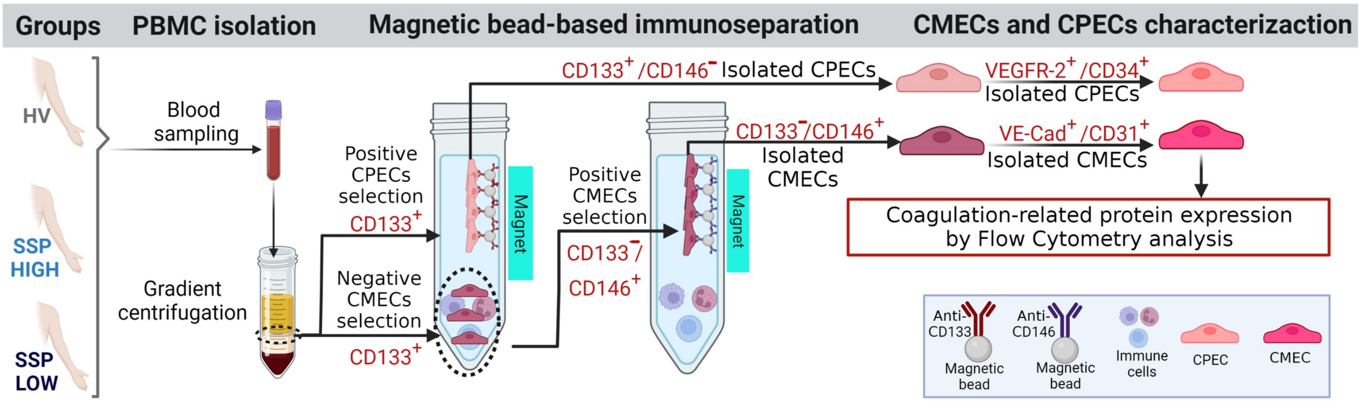

# Supplemental Figure S5

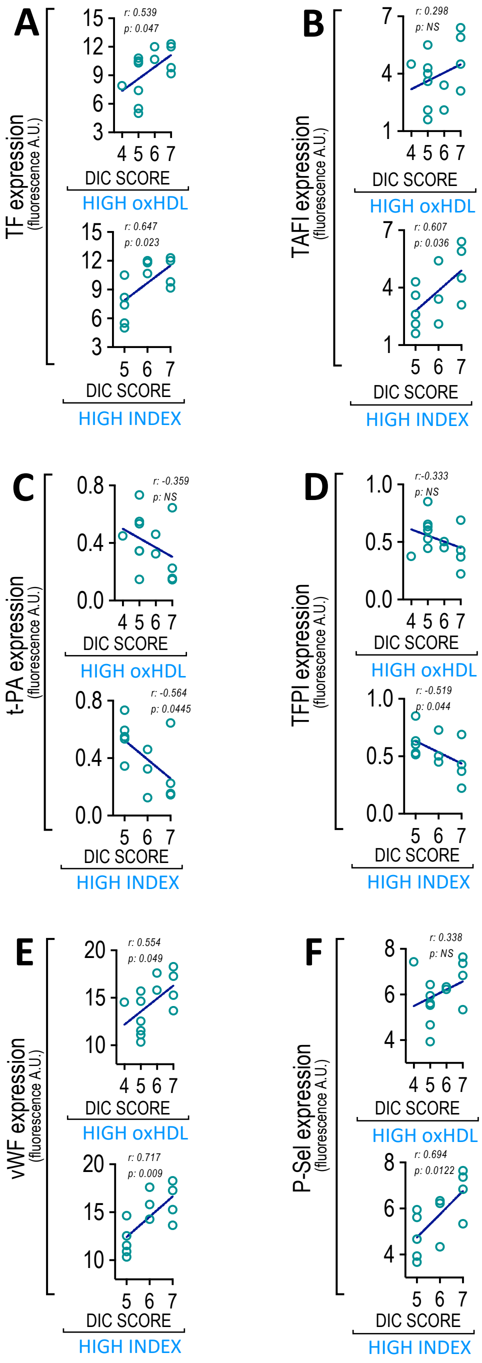

# Supplemental Figure S6

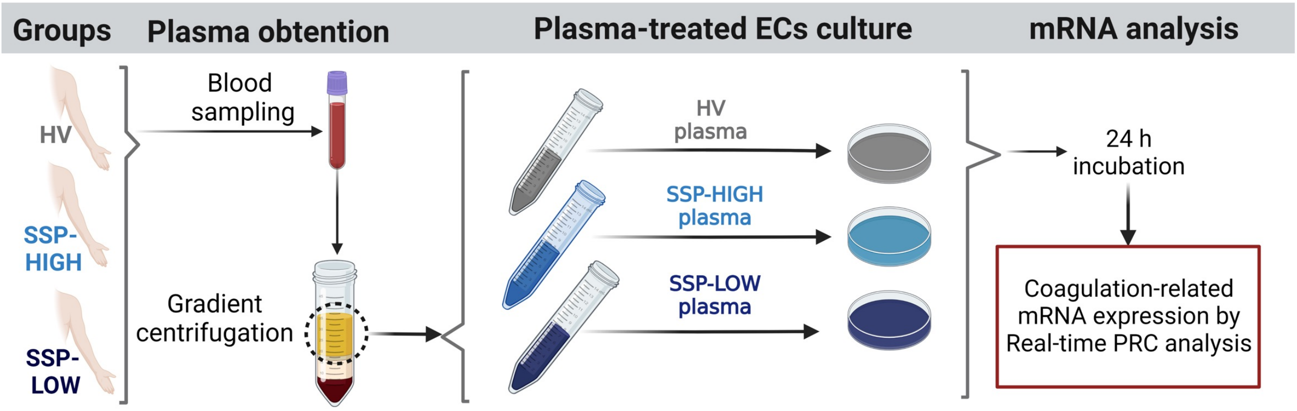

# Supplemental Figure S7

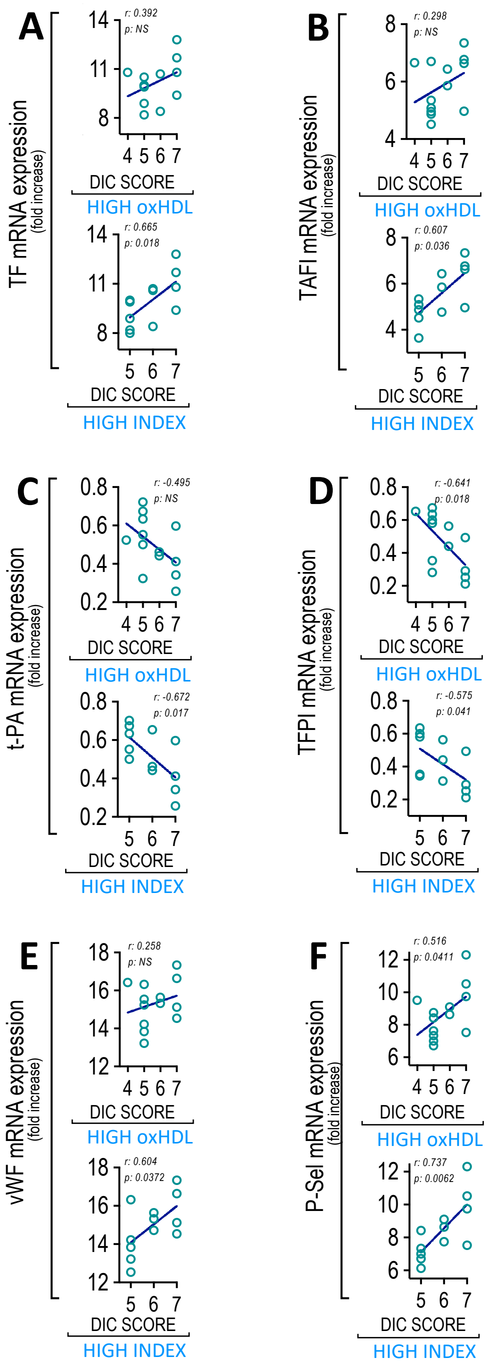

## Supplemental Figure S8

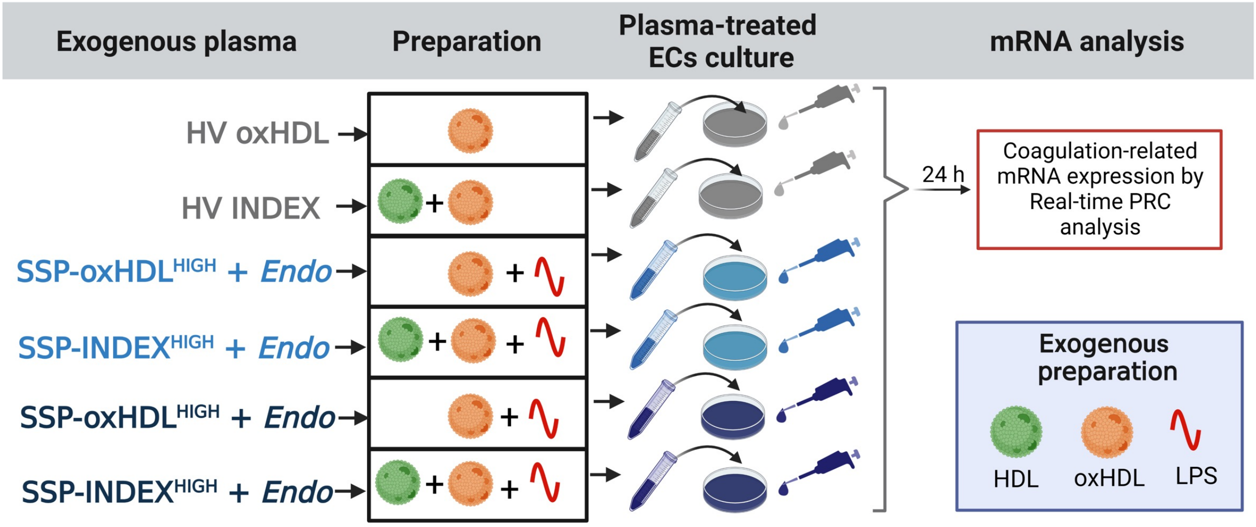

Supplement: Supplementary file 1 [file antioxidants-12-00543-s001.zip › antioxidants-2186699-supplementary.pdf]
